# Parental occupational exposure to organic solvents and autism spectrum disorder

**DOI:** 10.1192/j.eurpsy.2026.11168

**Published:** 2026-07-31

**Authors:** Y. Tebourbi, A. Chouchane, C. Mahjoub, H. Ben Abid, A. Gaddour, S. Ben Fredj, A. Aloui, I. Kacem, M. Makhloufi, M. Bouhoula, A. Brahem, H. Kalboussi, O. ElMaalel, C. Ben Hammouda, A. Guedria, S. Chatti

**Affiliations:** 1 Occupational Medicine and Professional Pathologies Department, Farhat Hached Teaching Hospital; 2 Faculty of Medicine of Sousse, Research Laboratory LR19SP03, University of Sousse, Sousse; 3 Child Psychiatry Department, Fattouma Bourguiba University Hospital, Monastir; 4 Occupational Medicine and Professional Pathologies Department, Ibn El Jazzar teaching Hospital, Kairouan; 5 Department of Epidemilogy, Farhat Hached Teaching Hospital, Sousse, Tunisia

## Abstract

**Introduction:**

The etiology of autism spectrum disorder (ASD) is multifactorial, involving both genetic and environmental factors. Parental occupational exposure to neurotoxicants such as organic solvents has been proposed as a potential environmental risk factor.

**Objectives:**

This study aimed to investigate the association between parental occupational exposure to organic solvents and the risk of ASD in Tunisian children.

**Methods:**

A case-control study was conducted between May and December 2022. Cases were children with a confirmed diagnosis of ASD, based on DSM-5 criteria who were compared to controls with no neuropsychiatric disorder. A pre-established questionnaire was used to collect sociodemographic and occupational data from parents. Occupational exposures lasting a minimum of one year and starting at least three months before conception, were considered relevant. Exposure to solvents was evaluated based on an exposure matrix.

**Results:**

A total of 70 ASD cases and 70 controls were included in the study. The mean age of the cases was 9.3 ± 3.0 years compared to 7.0 ± 2.8 years for the controls, with a statistically significant difference (p < 10ˉ^3^). The most common occupation among mothers in both groups was manual workers (63% in cases vs. 59.7% in controls, p = 0.71). Among mothers of cases, 21.4% were exposed to organic solvents [vs 38.6% in controls]. The handling of organic solvents by mothers was associated with a significantly lower risk of ASD (p=0.02; OR=0.43 95% CI [0.20-0.91]). The predominant occupation for fathers of cases was drivers (17.1%) and for controls was manual workers (20%). Paternal exposure to organic solvents was reported in 32.9% of cases [vs 31.4% in controls] with no significant association found between the father’s handling of organic solvents and the risk of ASD.

**Conclusions:**

In this study, only maternal occupational exposure to organic solvents was associated with a significantly lower risk of ASD occurrence. This unexpected protective association requires further investigation and may reflect study limitations.

**Disclosure of Interest:**

None Declared

